# 

**Published:** 1870-06

**Authors:** 


### Books and Pamphlets Received.

Surgical Memoirs of the War of the Rebellion. Collected and Published by the United States Sanitary Commission. By John A. Liddell, A. M., M. D. Edited by Prof. Frank Hastings Hamilton. New York: Hurd & Houghton for the U. S. Sanitary Commission. Received through Martin Taylor.

Obstetric Operations, Including the Treatment of Hemorrhage. By Robert Barnes, M. D., Lond., F. R. C. P., Obstetric Physician to, and Lecturer on Midwifery and the Diseases of Women and Children at St. Thomas' Hospital, etc. With additions by Benj. F. Dawson, M. D., late Lecturer on Uterine Pathology, in the Clinical Department of the University of New York. D. Appleton & Co. Received through Martin Taylor.

A Physician's Problems. By Charles Elam, M. D., M. R. C. P. Boston: Fields, Osgood & Co. 12mo. pp. 400.

The Men who Advertise: American Newspaper, Rate-Book and Directory, 1870. New York: Geo. P. Rowell & Co.

Notes on the Physiology and Pathology of the Nervous System with reference to Clinical Medicine. By Meredith Clymer, M. D. Reprinted from the New York Medical Journal for May and June, 1870.

Health officers' Annual Report of the City of Rochester, for the year ending March 31, 1870.

Constitution, By-Laws, Code of Ethics, and Fee-Bill, of the Medical Association of the City of Toledo, Ohio. Adopted March 11, 1870.

The American Grocer. A Weekly Trade Journal. John Darby & Co., 161 William street, New York.

The Little Corporal. Chicago: Sewell & Miller.

The Sunday School Workman. New York: Rev. Alfred Taylor.

The Writing Teacher. New York: A. M. Ellsworth & Co.

The Cape Ann Advertiser. Gloucester, Mass.

The Physician's Monitor for 1870.

Minutes of the Twenty-first Annual Meeting of the American Medical Association, held in the City of Washington May 3d, 4th, 5th, and 6th, 1870. W. B. Atkinson, M. D., Secretary.

